# Retraction: Effects of dexmedetomidine on postoperative delirium and expression of IL-1β, IL-6, and TNF-α in elderly patients after hip fracture operation

**DOI:** 10.3389/fphar.2024.1483859

**Published:** 2024-08-27

**Authors:** Frontiers Editorial Office

The journal retracts the 12 May 2020 article cited above.

Following publication, concerns were raised regarding the clinical trial registration for this study. An investigation was conducted in accordance with Frontiers’ policies, and it was confirmed that, contrary to the statements in the article, the study was not an accurate reflection of the study registered with the clinical trial registry. Lack of appropriate clinical trial registration is a breach of Frontiers’ guidelines and those of the Committee on Publication Ethics. As such, this article is being retracted. The authors received a communication regarding the retraction and had a chance to respond. This communication has been recorded by the publisher.

Frontiers would like to thank Prof. John Loadsman for contacting the journal regarding the published article.

